# A Case of Metastasis

**Published:** 1892-04

**Authors:** Wm. Steinrauf

**Affiliations:** St. Charles, Mo.


### A CASE OF METASTASIS.

WM. STEINRAUF, M. D., ST. CHARLES, MO.

Some months ago an unmarried lady, twenty-six years old, was brought to my office from a distant town, to be treated for a chest trouble—asthma, her allopathic physicians had called it. She has had this trouble now for about one year. Her pulse is one hundred and ten, and the temperature one hundred and one, very quick breathing all the time, and always worse at night; can sleep only a very short time, on account of this dyspnœa. There is a rattling noise on her chest, and a hard cough, with free expectoration.

After watching her symptoms for a little while, I came to the conclusion that there was chronic bronchitis, with asthma.

The remedies for a week or ten days did some little good. "I am better than I have been for a long time," were her own words. Still I was convinced that the disease had the best of me, and that I was not making much headway. Sulphur$^{dmm}$ was given now to clear up the case, and to re-develop any hidden trouble in the system. After three days her pulse was ninety, and her temperature ninety-nine. Cough, rattling, and the terrible dyspnœa were gone, and a most painful *ovarian neuralgia* had come on.

After some questioning it came to light that the lady about two years ago had been "sickly with her womb," as she expressed it. Several physicians had finally cured her of it, and after this scientific cure, in which vaginal injections of Carbolic acid and Sulphate of Zinc, etc., had played a prominent part, the chest trouble came on.

The indicated remedies, Cimicifuga and Lilium-tigrinum$^{dmm}$ soon cured the ovaritis, and she remains well to date.

